# Supplementary material for: Splice-Junction-Based Mapping of Alternative Isoforms in the Human Proteome
Source: Cell Rep. Author manuscript; Available in PMC 2020 Jan 15. (PMC6961840; doi:10.1016/j.celrep.2019.11.026)
Supplement: 2 [file NIHMS1546469-supplement-2.zip › DF1/PXD010154/Liver/Liver_52_CES1_TLKMSQPLLGTVIDGMLLLK.pdf]

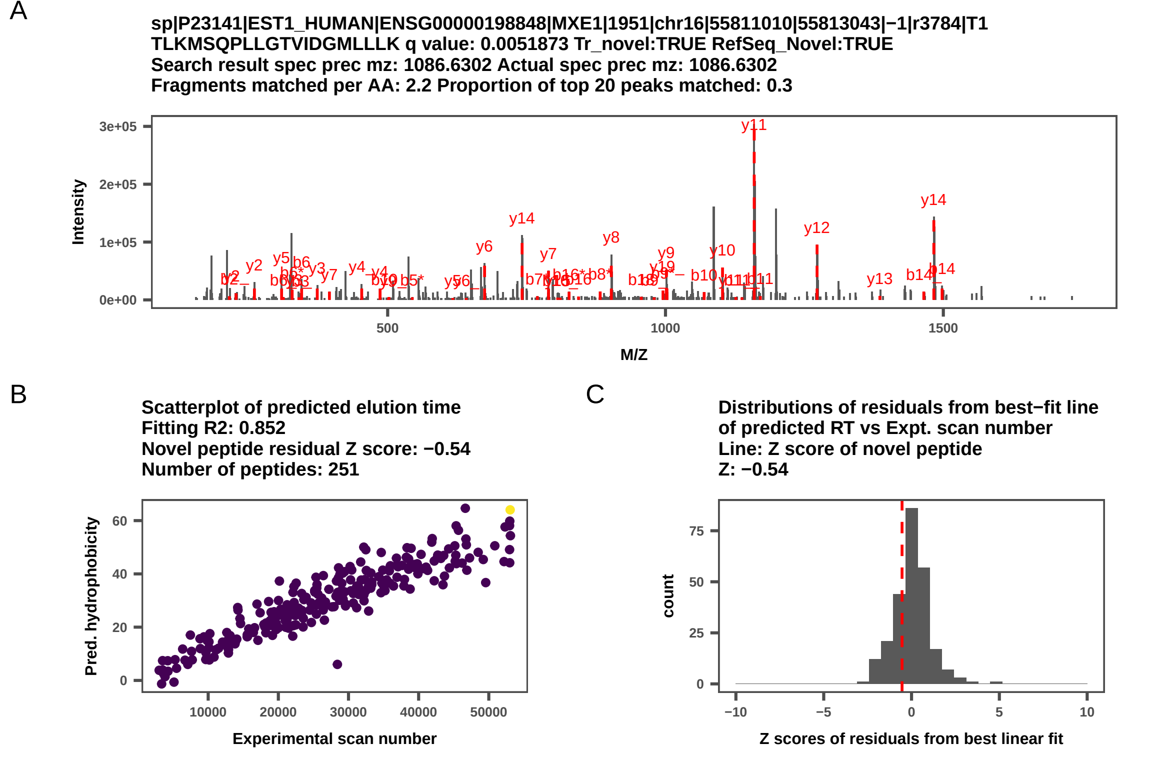
A
sp|P23141|EST1_HUMAN|ENSG00000198848|MXE1|1951|chr16|55811010|55813043|−1|r3784|T1
TLKMSQPLLGTVIDGMLLLK q value: 0.0051873 Tr_novel:TRUE RefSeq_Novel:TRUE
Search result spec prec mz: 1086.6302 Actual spec prec mz: 1086.6302
Fragments matched per AA: 2.2 Proportion of top 20 peaks matched: 0.3
Intensity
3e+05
2e+05
1e+05
0e+00
M/Z
500
1000
1500
y11
y14
y12
y14
y8
y6
y7
y9
y10
y2
y5
b6
y3
y7
y4
y13
b14
b14
b10
B
Scatterplot of predicted elution time
Fitting R2: 0.852
Novel peptide residual Z score: −0.54
Number of peptides: 251
Pred. hydrophobicity
0
20
40
60
Experimental scan number
10000
20000
30000
40000
50000
C
Distributions of residuals from best−fit line
of predicted RT vs Expt. scan number
Line: Z score of novel peptide
Z: −0.54
count
0
25
50
75
Z scores of residuals from best linear fit
−10
−5
0
5
10
